# The Benson Complex Figure Test for the Differential Diagnosis of Dementias

**DOI:** 10.3390/neurosci7020038

**Published:** 2026-03-20

**Authors:** Marina Papadogiani, Theodoros Fasilis, Akyllina Despoti, Vasiliki Kamtsadeli, Maria Hantzopoulou, Niki Tsinia, Evdoxia Lykou, Lina Chatziantoniou, Dimitrios Chousos, Kostas Siarkos, John D. Papatriantafyllou

**Affiliations:** 1 Third-Age Day Center IASIS, 16562 Athens, Greece; mpapadogianni@outlook.com (M.P.); fasilistheo@med.uoa.gr (T.F.); a.despoti@yahoo.com (A.D.); vkamtsadeli@gmail.com (V.K.); mariahatzopoulou@yahoo.gr (M.H.); evilykou@gmail.com (E.L.); lhatziantoniou@gmail.com (L.C.); chousos@hotmail.com (D.C.); 2 1st Department of Neurosurgery, Epilepsy Surgery Unit, Laboratory of Clinical Neuropsychology, National & Kapodistrian University of Athens, School of Medicine, Evaggelismos Hospital, 10676 Athens, Greece; 3 Clinical Ergospirometry, Exercise & Rehabilitation Lab, School of Medicine, National and Kapodistrian University of Athens, 11528 Athens, Greece; 4 1st Psychiatric Clinic, Eginition Hospital, School of Medicine, National and Kapodistrian University of Athens, 11528 Athens, Greece; ntsinia@gmail.com; 5 Third Age Day Center IASIS, 24131 Kalamata, Greece; kosia2006@yahoo.co.uk; 6 1st Neurological Clinic, Eginitio Hospital, School of Medicine, National and Kapodistrian University of Athens, 11528 Athens, Greece; 7 Memory Clinic, Medical Center of Marousi-Athens, School of Medicine, National and Kapodistrian University of Athens, 11528 Athens, Greece

**Keywords:** Benson Complex Figure Test, dementia, visuospatial impairment, cognitive assessment, differential diagnosis

## Abstract

The Benson Complex Figure Test (BCFT) is a neuropsychological tool designed to assess visuospatial construction and visual memory with lower complexity than traditional tests. This study evaluated its ability to differentiate between major dementia subtypes. In a retrospective cross-sectional analysis of 1428 participants from a Greek third-age day center (healthy participants [Controls]; patients diagnosed with Alzheimer’s disease dementia [ADD], Lewy body dementia [LBD], Frontotemporal dementia [FTD: behavioral variant (BV), non-fluent variant (NFV), semantic variant (SV)], Corticobasal dementia [CBD], Parkinson’s disease dementia [PDD], and mixed Cardiovascular dementia with Alzheimer’s disease [CVD/AD]), all participants completed the BCFT and the Mini-Mental State Examination (MMSE). Multinomial logistic regression, adjusted for age, sex, and education, revealed distinct BCFT profiles across dementia subtypes. Patients with CBD showed significantly lower copy scores than those with ADD (*p* = 0.006). The FTD-NFV group exhibited superior memory scores compared to all other dementia subtypes (*p* < 0.001). Poorer BCFT recognition performance was strongly associated with diagnoses of ADD (OR = 0.39, *p* = 0.012), FTD-BV (OR = 0.22, *p* = 0.025), and PDD (OR = 0.26, *p* < 0.001). Classification accuracy was highest for controls and ADD (sensitivity > 89%) but low for rarer subtypes (<25%), partly reflecting sample size limitations. In conclusion, the BCFT captures distinct visuospatial and memory profiles across dementia syndromes, supporting its potential utility in differential diagnosis, particularly for common subtypes such as ADD. Its simpler design may facilitate assessment in older adults, although validation in larger and more balanced cohorts is required for rarer dementias.

## 1. Introduction

Dementia is a growing global health concern, affecting over 55 million people, and this figure is projected to triple by 2050 [1,2]. As dementia prevalence grows, understanding its multifaceted impact becomes critical for developing effective interventions and support mechanisms.

One of the key cognitive domains affected by dementia is visuoconstructional skills and visual memory [3,4]. These skills, which involve the ability to perceive, analyze, and interpret visual information about spatial relationships, are essential for everyday tasks such as navigating environments, recognizing objects and shapes, and performing complex motor activities like cooking or driving [5]. In individuals with dementia, impairments in visuospatial functioning can manifest early and progressively worse, severely impacting their independence and quality of life [6,7].

The extent and nature of visuospatial deficits in dementia vary depending on the type of dementia. Alzheimer’s disease (AD) is associated with early visuospatial deficits, which have been linked to structural and functional alterations in the parietal lobe [4,7]. Poor copy performance in AD is significantly related to atrophy of the right temporoparietal cortex, involving spatial perception and attention [6,8,9]. These impairments play a crucial role in distinguishing AD from other forms of dementia [9]. Many studies have confirmed that complex figure tests have good prognostic and diagnostic potential for AD [10,11,12].

Lewy body dementia (LBD) is uniquely associated with pronounced visuoconstructional deficits, often appearing early in the disease course [13]. The individuals in the early stages of disease exhibit significant impairments in visuoconstructional abilities, while their memory remains relatively preserved [14]. Other tests of figure copying including Rey–Osterrieth Complex Figure Test (ROCFT) [15] and Bender Gestalt Test [16,17] have shown to be impaired in LBD and this impairment correlates with occipital cortex hypometabolism [18].

In Parkinson disease (PD), the dual-syndrome hypothesis divides executive dysfunction into a subtype implicating fronto–striatal circuits, and a posterior/visuospatial subtype [19,20]. Visuospatial impairment is an early feature of PD and figure copying tests have been shown to be prone to the disease [19,21].

Furthermore, frontotemporal dementia (FTD) usually is marked by behavioral changes (behavioral variant—FTD-BV), and language impairments either in the form of speech production (primary progressive aphasia non-fluent/agrammatic variant—FTD-NFV), often accompanied by difficulties in executive functioning and social cognition [22,23], or deficits in word and object meaning (semantic variant—FTD-SV) [24]. However, episodic memory and visuospatial skills tend to remain largely unaffected [25]. Patients with FTD particularly those with FTD-BV, may display disorganized or fragmented drawings due to executive dysfunction rather than memory impairment [26]. These differences underscore the need for a nuanced understanding of how different types of dementia influence visuospatial abilities [25].

In corticobasal dementia (CBD), the visuoconstructive abilities often compromised due to asymmetric cortical and subcortical abnormalities, primarily affect the peri-Rolandic, parietal, and basal ganglia structures [27]. These structural changes disrupt the integration of visual and motor information, leading to difficulties in visuospatial tasks [28].

In cardiovascular dementia (CVD), several studies report that people commonly show impairments in visuoconstructional tasks [29]. These impairments are part of a broader pattern of cognitive deficits that also include executive dysfunction, attention problems, and memory issues. Some research suggests that visuoconstructive problems are not always stronger or more severe in CVD than in AD, especially on complex figure copying, while others showed variable patterns depending on task and subtype [30].

Understanding the potential differences in visuospatial and visuoconstructional deficits across dementia types is of paramount importance. Such insights not only contribute to more accurate diagnoses but also inform the creation of specialized rehabilitation programs. 

Thus, the aim of the present study is to investigate potential differences in visuospatial skills in major neurodegenerative disorders that give rise to distinct dementia syndromes (ADD, LBD, FTD [SV, BV, NFV], CBD, PDD, CVD/AD) in order to further identify distinct visuospatial profiles associated with each subtype and provide valuable insights into the underlying mechanisms of visuospatial decline.

## 2. Methods

### 2.1. Design

The study followed a retrospective cross-sectional design using previously collected clinical and neuropsychological data. The design focused on examining whether the BCFT (Total, Copy, Memory, Recognition) differs across dementia diagnoses and whether these scores are associated with diagnostic likelihood after statistical adjustment. As such, to evaluate the ability of BCFT performance to predict diagnostic subgroup involvement, a multinomial logistic regression model was fitted with diagnosis as a multi-class outcome and BCFT score as the primary predictor, with demographics (age, education, sex) as additional covariates. The dataset was complete, with no missing values for any of the analyzed variables.

Clinical diagnoses were established in routine practice by specialist neurologists and neuropsychiatrists according to established international criteria (ICD-11). Diagnostic work-up included clinical history, neurological examination, and standardized neuropsychological assessment, with neuroimaging and ancillary investigations used when clinically indicated. BCFT administration and scoring were performed by trained psychologists who were blinded to diagnostic group.

### 2.2. Participants

The sample was secondary and consisted of 1428 participants (65.7% women), with mean age 70.1 years (SD = 11.5 years). Mean educational years was 11 years (SD = 4.7 years). The participants were exclusively of Greek origin with Greek as their mother tongue. The data were collected from the medical records of the third-age day center IASIS located in Athens, Greece. The sample included healthy participants as well as patience groups diagnosed with ADD, PDD, LBD, CBD, FTD-BV, FTD-NFV, FTD-SV, and CVD/AD. Details concerning the sample are presented in Table 1.

### 2.3. Inclusion and Exclusion Criteria

Participants included in the study were adults aged 50 years or older, diagnosed with ADD, PDD, LBD, CBD, or FTD based on established clinical criteria. Healthy individuals without cognitive impairment were also included as controls. All participants were native Greek speakers.

Exclusion criteria comprised individuals with a history of stroke, traumatic brain injury, or other neurological disorders unrelated to neurodegenerative diseases. Participants with psychiatric disorders such as major depression or schizophrenia, which could confound cognitive assessment results, were also excluded. Lastly, severe sensory impairments, including uncorrected vision or hearing loss that could interfere with test performance, were also excluded.

### 2.4. Ethical Considerations

To ensure ethical conduct, the research was completed in accordance with the Helsinki Declaration, and participants provided informed consent by signing the Information and Declaration of Consent Regarding Personal Data. This form included an option for the patient and/or caregiver to provide permission for the center to use the patient’s data for research and educational purposes, which in turn provided us with relevant permission to access these data, by the center’s Ethics Committee (20 January 2021: Approval No. 01). Participants retained the right to withdraw from the study at any time, upon request.

### 2.5. Measurement Tools

The tools used in the research were the Mini-Mental State Examination (MMSE) and the BCFT. In more detail:

1. The global cognitive status of the participants was measured with the MMSE [31], scaled in Greek in 2000 [32]. The MMSE is a simplified, scored cognitive test and is divided into two sections, the first requiring only vocal responses and covering orientation, memory, and attention with a maximum score of 21 and the second section examining the ability to name, follow spoken and written commands, write a sentence spontaneously and copy a complex polygon similar to a Bender–Gestalt shape, with a maximum score of 9. The maximum score of the tool is 30 points and there is no specific time required for its completion.
2. The BCFT, which is a simplified version of the ROCFT [10], was used to measure participants’ visual constructional ability and visual memory. The BCFT is part of the Uniform Data Set, which was implemented in 2005 by the National Institute on Aging to measure cognitive performance in dementia and mild cognitive impairment due to AD [32]. The individual copies the figure and is then informed that they will be asked to do it from memory. The interval between copy and recall of the figure should be 10–15 min. After the delayed recall, the individual is asked to recognize the original stimulus from four similar figures. The figure is made up of 8 elements and two points are awarded if each element is correctly positioned and correctly shaped. If the figure is correctly designed, the maximum score is 17 points (1 point bonus).

### 2.6. Procedure

The medical files of the patients were collected from 2008 to 2022, and patients diagnosed with ADD, LBD, PDD, CBD, FTD (BV, NFV, SV), and CVD/AD were taken into account. It is important to note that we used secondary data, meaning that the sample did not undergo any specific procedures during the research, but data were obtained during their regular check-up at the premises of the third-age day center IASIS.

### 2.7. Statistical Analysis

Quantitative variables were expressed as mean values and standard division (SD). Primary analyses employed multinomial logistic regression to model diagnostic group classification. The full model examined the predictive utility of BCFT total, copy, memory, and recognition scores while controlling for demographic covariates. The control group was used as the reference category.

Model fit was assessed using −2 log likelihood, McFadden’s pseudo-R^2^, and Nagelkerke’s pseudo-R^2^. Likelihood ratio tests were conducted using Type II tests to evaluate the overall contribution of each predictor. Classification performance was evaluated through overall accuracy, confusion matrix, and per-class specificity and sensitivity. For interpretability, regression coefficients were converted to odds ratios (ORs) with 95% confidence intervals.

All statistical analyses were conducted using R statistical software (version 4.5.2). All reported *p*-values are two-tailed. Statistical significance was set at α = 0.05 (CI 95%).

## 3. Results

### 3.1. Descriptive Analysis

Across the diagnostic groups, Normal controls consistently achieved the highest scores in total BCFT (Mean = 29.62), copy (16.18), memory (12.78), and recognition (0.93) domains. In contrast, CBD and ADD patients exhibited the poorest total and copy performance, while ADD and CBD also demonstrated the lowest memory scores. Except the control group, FTD-NFV (0.70) and LBD (0.53) demonstrated the highest BCFT recognition scores, and the lowest were FTD-BV (0.34), FTD-SV (0.34), and CVD/AD (0.38). Descriptive details concerning the neuropsychological tools are presented in Table 2.

### 3.2. Assumption Check

Multicollinearity was assessed using tolerance statistics (1/variance inflation factors). All predictors showed acceptable collinearity (tolerance > 0.4), indicating no violation of regression assumptions, as seen in Table 3, Table 4, Table 5 and Table 6.

### 3.3. Multinomial Logistic Regression

According to the data in Table 7, the model concerning BCFT total score showed particularly strong negative coefficients for SV (β = −0.592), CBD (β = −0.617), and CVD/AD (β = −0.566), suggesting substantial discrimination ability. When covariates (age, education, sex) were included in the model, BCFT remained a highly significant predictor for all diagnostic groups (*p* < 0.001). The full model demonstrated improved fit with a substantially lower residual deviance (2388.44) compared to the simple model (2735.43). Age emerged as a significant covariate for several groups such as CVD/AD (β = 0.200, *p* < 0.001), ADD (β = 0.144, *p* < 0.001), CBD (β = −0.109, *p* < 0.001), LBD (β = 0.206, *p* < 0.001), PDD (β = 0.087, *p* < 0.001), NFV (β = −0.070, *p* = 0.033), and SV (β = −0.057, *p* = 0.026). Sex was significantly associated with several diagnostic groups like CVD/AD (β = −0.839, *p* = 0.006), NFV (β = −1.296, *p* = 0.048), and PDD (β = −0.915, *p* = 0.004). Education showed significant effects for ADD (β = −0.116, *p* < 0.001), CBD (β = −0.183, *p* = 0.006), CVD/AD (β = −0.095, *p* = 0.006), and SV (β = −0.219, *p* < 0.001).

The full model incorporating BCFT copy score with demographic covariates (age, education, sex) demonstrated strong predictive utility across all diagnostic groups. BCFT remained a highly significant predictor (*p* < 0.001) for all eight disease groups, with particularly strong negative coefficients for CBD (β = −0.523, *p* < 0.001), BV (β = −0.434, *p* < 0.001), SV (β = −0.453, *p* < 0.001), and NFV (β = −0.428, *p* < 0.001), indicating substantial discrimination ability. Age emerged as a significant covariate for multiple groups including CVD/AD (β = 0.260, *p* < 0.001), LBD (β = 0.263, *p* < 0.001), ADD (β = 0.205, *p* < 0.001), PDD (β = 0.134, *p* < 0.001), and BV (β = 0.059, *p* = 0.002), while showing protective effects for CBD (β = −0.049, *p* = 0.056, trending toward significance). Sex was significantly associated with CVD/AD (β = −0.533, *p* = 0.031) and PDD (β = −0.656, *p* = 0.025), with females (SEX2) showing lower odds of these conditions relative to males. Education showed significant negative effects for SV (β = −0.244, *p* < 0.001), CBD (β = −0.196, *p* < 0.001), ADD (β = −0.158, *p* < 0.001), CVD/AD (β = −0.132, *p* < 0.001), and BV (β = −0.120, *p* < 0.001), indicating that higher education was protective across most diagnostic groups.

The BCFT memory model with demographic covariates (age, education, sex) demonstrated exceptional predictive utility across all diagnostic groups. BCFT showed particularly strong negative coefficients for ADD (β = −0.714, *p* < 0.001), BV (β = −0.667, *p* < 0.001), CBD (β = −0.667, *p* < 0.001), and SV (β = −0.677, *p* < 0.001), indicating robust discrimination ability, with odds ratios ranging from 0.489 to 0.513 for these groups, representing substantial risk reduction with higher BCFT memory scores. Age emerged as a significant covariate for multiple groups including CVD/AD (β = 0.189, *p* < 0.001), LBD (β = 0.195, *p* < 0.001), ADD (β = 0.130, *p* < 0.001), and PDD (β = 0.083, *p* < 0.001), while showing protective effects for CBD (β = −0.115, *p* < 0.001) and SV (β = −0.067, *p* = 0.011). Sex was strongly associated with several groups including CVD/AD (β = −1.119, *p* < 0.001), PDD (β = −1.037, *p* < 0.001), NFV (β = −1.461, *p* = 0.025), and BV (β = −0.917, *p* = 0.017), with females (SEX2) showing substantially lower odds of these conditions. Education showed significant negative effects for SV (β = −0.246, *p* < 0.001), CBD (β = −0.222, *p* < 0.001), ADD (β = −0.136, *p* < 0.001), BV (β = −0.105, *p* = 0.019), and CVD/AD (β = −0.114, *p* = 0.002), indicating consistent protective effects across most diagnostic groups.

Lastly, the model incorporating BCFT recognition score with demographic covariates demonstrated exceptionally strong predictive effects, with BCFT showing dramatically large negative coefficients across all diagnostic groups. BCFT was a highly significant predictor (*p* < 0.001) for all eight disease groups, with particularly extreme coefficients for BV (β = −3.052, *p* < 0.001), CBD (β = −3.121, *p* < 0.001), SV (β = −2.667, *p* < 0.001), ADD (β = −2.541, *p* < 0.001), and CVD/AD (β = −2.557, *p* < 0.001), representing odds ratios as low as 0.044 to 0.079, indicating that higher BCFT scores confer very substantial protective effects. Age emerged as a significant covariate for multiple groups including CVD/AD (β = 0.248, *p* < 0.001), LBD (β = 0.251, *p* < 0.001), ADD (β = 0.191, *p* < 0.001), PDD (β = 0.126, *p* < 0.001), and BV (β = 0.044, *p* = 0.020), while showing protective effects for CBD (β = −0.058, *p* = 0.012). Sex was significantly associated with CVD/AD (β = −0.733, *p* = 0.003) and PDD (β = −0.790, *p* = 0.007), and showed near-significant effects for BV (β = −0.594, *p* = 0.064) and NFV (β = −1.179, *p* = 0.061), with females showing lower odds of these conditions. Education showed significant negative effects for SV (β = −0.264, *p* < 0.001), CBD (β = −0.230, *p* < 0.001), ADD (β = −0.167, *p* < 0.001), CVD/AD (β = −0.144, *p* < 0.001), LBD (β = −0.151, *p* = 0.004), and BV (β = −0.127, *p* < 0.001), indicating consistent protective effects across most diagnostic groups.

### 3.4. Model Performance

For BCFT total score, the confusion matrix revealed an overall classification accuracy of 72.4%. The model showed particularly high sensitivity for Control (97.5%) and ADD (89.6%) groups, but lower sensitivity for other disease groups. However, several groups (BV, CBD, CVD/AD, LBD, NFV, PDD, SV) showed low sensitivity (<25%), indicating poor classification for these categories. For BCFT copy, the overall accuracy is 66.11%, for BCFT memory 73.28%, and for BCFT recognition 67.48%. Specifically, the model focusing on the BCFT copy achieved substantial explanatory power (Nagelkerke R^2^ = 0.633). For BCFT memory, the model achieved excellent explanatory power (Nagelkerke R^2^ = 0.766, McFadden’s R^2^ = 0.444). For the BCFT recognition, the model achieved substantial explanatory power (Nagelkerke R^2^ = 0.630, McFadden’s R^2^ = 0.313). The details are presented in Table 8.

According to Table 9, the models for BCFT significantly outperformed the null model, with total score (LR χ^2^(8) = 689.87, *p* < 0.001), copy score (LR χ^2^(8) = 229.52, *p* < 0.001), memory score (LR χ^2^(8) = 762.90, *p* < 0.001), and recognition (LR χ^2^(8) = 220.12, *p* < 0.001).

The classifier demonstrated strong discriminatory performance for the Control and ADD groups but failed to reliably identify most of the rarer dementia subtypes, as shown by per-class sensitivity (Table 10) and specificity (Table 11). The model excelled at identifying healthy controls, with sensitivity ranging from 0.929 to 0.984 across the four test conditions (total, copy, memory, recognition). Specificity for the Control class was also high, between 0.785 and 0.887. Performance for ADD was also robust, with sensitivity consistently high (0.788 to 0.911) and specificity moderate to good (0.712 to 0.744). Critically, the model showed near-zero sensitivity for several conditions like LBD (0.000), NFV (0.000), and PDD (0.000). While specificity for these classes was perfect (1.000), this indicates that the model almost never predicted them, effectively classifying all such cases as another condition. CBD and CVD/AD showed very low but non-zero sensitivity (0.019–0.227 for CBD; 0.020–0.054 for CVD/AD), despite high specificity (0.989). BV and SV were detected with minimal success, with BV sensitivity peaking at 0.058 and SV at 0.069.

## 4. Discussion

The present study examined visuospatial and visuoconstructional abilities across multiple dementia subtypes, highlighting distinct neuropsychological profiles that may inform differential diagnosis and targeted interventions [10,11,12,25]. CBD participants exhibited severe copy and memory impairments, consistent with prior findings linking peri-Rolandic and parietal cortical atrophy, basal ganglia dysfunction, and tau pathology to disrupted visuomotor integration and constructional apraxia [27,33,34]. These deficits likely reflect impaired connections between parietal and motor regions, resulting in disorganized spatial representations, while recognition performance remained relatively preserved, indicating that memory systems were less affected [10,12].

Participants belonging to the FTD-SV group showed copy and memory deficits, which may arise from semantic processing difficulties affecting object recognition rather than pure spatial impairment [22,23,24,25]. FTD-BV patients showed disorganized or fragmented copies alongside recognition deficits, consistent with executive dysfunction stemming from frontal white matter tract abnormalities, which disrupt planning and organization rather than visuospatial perception per se [22,26]. In FTD-NFV, the predominant cognitive deficits are effortful, agrammatic speech production, phonemic paraphasias, impaired syntax, and reduced fluency, with relatively preserved visuospatial recognition and memory, at least early in the disease course [35].

Participants diagnosed with PDD demonstrated deficits in most scores but relatively preserved recognition, aligning with the dual-syndrome hypothesis in which posterior cortical involvement selectively impairs visuospatial processing, while medial temporal memory networks remain largely intact [19,20,21]. These visuoconstructional deficits may also be exacerbated by dopaminergic and cholinergic dysfunction affecting visual attention and discrimination, with variability influenced by disease stage [19,21].

LBD participants showed pronounced memory deficits but comparatively intact copying, reflecting posterior cortical and occipital hypometabolism that primarily disrupts memory consolidation and attentional processes [13,14,15,18,36]. Recognition performance in LBD was unexpectedly similar to controls, potentially influenced by disease stage and compensatory mechanisms such as visual hallucinations, highlighting the heterogeneity of visuospatial and memory deficits in this group [14,15].

The ADD group displayed prominent recognition deficits with relatively preserved copy performance, consistent with medial temporal lobe and hippocampal pathology disrupting memory encoding and retrieval, while early-stage posterior cortical atrophy may have limited effects on figure copying [4,7,8,12]. These findings reinforce the notion that visuospatial deficits in AD progress gradually, becoming more pronounced as posterior cortical regions degenerate, leading to higher-order visual impairments such as simultanagnosia and environmental agnosia [4,6,8].

In CVD, cognitive findings typically include visuospatial impairments, executive dysfunction, slowed processing speed, and difficulties with attention and visuomotor integration, reflecting ischemic injury to subcortical and cortical networks involved in visual-spatial and attentional processing [37]. When CVD coexists with AD, cognitive profiles often reflect a combination of visual–spatial and executive deficits attributable to profound memory and visuoperceptual dysfunction, leading to a broader and more severe pattern of visual and cognitive impairment than seen in either condition alone [7,12,37]. This result mirrors the profile of the ADD group, where prominent deficits in memory and recognition were also evident.

In examining key points and discrepancies, we found that FTD-SV patients scored lower than expected concerning the BCFT total score. This may reflect sample characteristics, age, or disease severity. Conversely, LBD patients scored higher than some of the literature reports [2,4,9,15,24,27,33], potentially due to a milder disease stage or sample selection. On the copy subscale, healthy controls performed best (16), followed by PDD (13), a cluster of FTD-BV, FTD-NFV, ADD, CVD/AD, and LBD showed intermediate performance (12), FTD-SV scored lower than these groups (10), and CBD had the lowest performance overall (8.2). This pattern aligns with the literature. According to review studies [5,10,11,12,17,18], figure copy tasks indicate significant associations with the posterior parieto–temporo–occipital cortex, especially in dementia, underscoring the importance of these regions in encoding and organizing complex visual stimuli into a coherent representation. On the memory subscale, FTD-NFV showed moderate impairment (8), CVD/AD, LBD, CBD, and FTD-SV clustered at low performance levels (3–3.6), and ADD demonstrated the lowest memory scores overall (2.5). Both lesion and functional imaging studies [4,7,8,14,18,25] confirm that effective delayed recall engages networks rooted in temporal and parietal cortices, consistent with the profound memory impairments seen in ADD and related syndromes. For the recognition subscale, FTD-NFV showed mild impairment (0.7), PDD and LBD had moderate deficits (0.5), ADD performed worse (0.4), and FTD-SV, FTD-BV, and CBD showed the lowest recognition performance (0.3). Recognition taps familiarity and recollection processes that are supported by medial temporal structures (including hippocampal and parahippocampal regions) and their interactions with parietal and frontal cortices; these processes are distinct yet overlapping with those in delayed recall (memory subscale), with recognition relying more on preserved encoding (copy subscale) and storage mechanisms [6,8,12,14,18,25].

Overall, these findings largely align with the international literature. Posterior cortical and medial temporal pathology (as in ADD and CBD) is associated with the most pronounced deficits in figure copying, delayed memory, and recognition [4,6,8,10,18,33,34], while frontal and linguistic variants of FTD show relatively preserved total and copy scores but memory and recognition impairments [8,12,24,25,35]. LBD shows notable memory deficits with relatively less severe copy and recognition deficits compared to typical ADD [3,14,15,16,17,18], while CBD manifests pronounced copy and memory difficulties with heterogeneous memory effects reflecting distributed cortical dysfunction [6,27,33,34].

In summary, the distinct cognitive profiles revealed by the copy, memory, and recognition subscales can provide valuable support for differential diagnosis among dementia subtypes. For instance, pronounced deficits in figure copying and delayed memory are indicative of CBD, ADD, CVD/AD, LBD and FTD-SV or posterior cortical involvement, whereas relatively preserved visuospatial construction and recognition combined with intermediate memory impairment point toward frontal or linguistic variants of FTD. LBD and PDD can be distinguished by the presence of prominent recognition deficits with less severe memory impairment, while CBD is characterized by severe constructional difficulties and heterogeneous memory and recognition performance.

The strengths of this study include the large sample size and the use of validated neuropsychological tools. However, certain limitations must be acknowledged. The most significant stems from the considerable disparity in group sizes among the different dementia subtypes. While the total sample size is a strength, this overall number masks a critical imbalance. Our cohort includes a very large group of participants with ADD and a substantial control group, which provides robust data for these groups. However, several key diagnostic groups, crucial for the differential diagnosis focus of our paper, are represented by much smaller sample sizes (specifically the groups for LBD, n = 23; CBD, n = 22; NFV, n = 11) containing fewer than 30 participants. In addition, there is a mismatch between the gender ratio and the age structure of the groups being compared, as seen in Table 1. Counterbalancing of these elements followed the recommendations for multinomial logistic regression designs, as implemented in our statistical approach. Furthermore, the reliance on secondary data introduces constraints, particularly regarding the control of confounding variables such as medication effects and disease progression. Additionally, the study sample consisted exclusively of Greek participants, necessitating further research to confirm the generalizability of these findings to other populations.

Future research should explore longitudinal changes in BCFT performance to establish its predictive validity for cognitive decline, and combine BCFT results with neuroimaging biomarkers to further elucidate the neural substrates underlying these visuospatial and memory deficits [38]. Additionally, larger samples of less prevalent dementias, such as CBD and LBD, will help refine the sensitivity and specificity of BCFT measures across neurodegenerative disorders.

## 5. Conclusions

The present study demonstrates that visuospatial and visuoconstructional performance differs markedly across major dementia syndromes. The most profound deficits were observed in CBD, ADD, CVD/AD, LBD and FTD-SV, consistent with disruptions in posterior parietal, occipital, and subcortical visuospatial networks. PDD and FTD-NFV patients showed relatively preserved or only moderately impaired visuoconstructional abilities once global cognition was accounted for, highlighting the importance of disease severity and subtype-specific neuroanatomical involvement. Taken together, copy skills were most severely impacted in CBD, followed by FTD-SV, while ADD, CVD/AD, LBD, FTD-BV, and FTD-NFV showed intermediate impairment, with PDD being less affected. Memory deficits were most pronounced in ADD, followed by CVD/AD, LBD, CBD, and FTD-SV, with FTD-NFV showing moderate impairment and PDD the least affected. Regarding recognition, the most prominent deficits were observed in FTD-SV, FTD-BV, and CBD, followed by ADD and CVD/AD, with LBD and PDD showing moderate impairment and FTD-NFV the mildest deficits. 

Incorporating comprehensive visuospatial assessment into standard neuropsychological batteries may aid earlier differentiation, refine clinical profiling, and support more targeted intervention strategies. Future work integrating neuroimaging, biomarkers, and longitudinal follow-up will be crucial to clarifying the neural mechanisms underlying these cognitive patterns and to validating these markers across diverse populations.

## Figures and Tables

**Table 1:** Sample demographics, diagnosis, and MMSE scores.

	Total Sample		Diagnosis							
		Control	ADD	CVD/AD	LBD	FTD-BV	FTD-NFV	FTD-SV	CBD	PDD
	n = 1428; 100%	n = 674 47.20%	n = 405 28.36%	n = 149 10.43%	n = 23 1.61%	n = 52 3.64%	n = 11 0.77%	n = 29 2.03%	n = 22 1.54%	n = 63 4.41%
Sex										
Men	487 (34.1)	208 (30.7)	118 (34.8)	74 (49.6)	7 (30.4)	23 (42.9)	6 (64.7)	12 (41.9)	5 (22.7)	34 (56.7)
Women	941 (65.9)	466 (69.3)	287 (65.2)	75 (50.4)	16 (69.6)	29 (57.1)	5 (35.3)	17 (58.1)	17 (77.3)	29 (43.3)
Age (years), mean (SD)	70.1 (11.5)	62.9 (10.0)	78.2 (6.9)	80.3 (5.7)	80.5 (6.4)	69.8 (9.6)	63.7 (8.4)	66.9 (7.3)	61.7 (7.5)	74.3 (7.5)
Educational years, mean (SD)	11.0 (4.7)	13.4 (3.5)	8.4 (4.6)	8.9 (4.4)	8.6 (4.3)	10.2 (4.2)	12.3 (4.9)	8.5 (4.7)	9.3 (3.4)	11 (4.2)
Duration (months), mean (SD)	19.2 (24.0)	N/A	35.7 (20.8)	33.1 (11.4)	32.6 (17.0)	35.5 (15.8)	35.5 (18.4)	32.6 (14.8)	30.6 (15.8)	44.9 (34.4)
MMSE, mean (SD)	24.3 (6.7)	29.2 (0.9)	19.7 (6.2)	20.3 (5.9)	20.4 (5.8)	19.7 (6.8)	21.7 (9.6)	17.2 (9.1)	17.6 (9)	25.5 (4.5)

**Table 2:** Descriptive statistics concerning BCFT scores among the groups.

Diagnosis (n)	BCFT Total			BCFT Copy			BCFT Memory			BCFT Recognition		
	Mean (SD)	Min	Max	Mean (SD)	Min	Max	Mean (SD)	Min	Max	Mean (SD)	Min	Max
Control (674)	29.62 (3.453)	19	35	16.18 (1.979)	12	17	12.78 (2.933)	0	17	0.93 (0.248)	0	1
ADD (414)	14.24 (6.724)	0	30	12.19 (5.282)	0	17	2.54 (3.262)	0	14	0.42 (0.495)	0	1
CVD/AD (149)	14.77 (6.766)	0	31	12.04 (4.898)	0	17	3.36 (3.710)	0	14	0.38 (0.486)	0	1
LBD (23)	16.92 (6.530)	2	27	12.96 (4.463)	1	17	3.64 (3.119)	0	10	0.53 (0.507)	0	1
FTD-BV (52)	16.36 (7.928)	1	31	12.54 (5.412)	0	17	4.21 (4.291)	0	13	0.34 (0.481)	0	1
FTD-NFV (11)	19.00 (11.275)	1	32	12.27 (7.484)	0	17	8.00 (5.646)	0	17	0.70 (0.483)	0	1
FTD-SV (30)	14.29 (8.090)	1	31	10.21 (6.379)	0	17	3.12 (3.804)	0	14	0.34 (0.481)	0	1
CBD (22)	12.38 (9.504)	1	30	8.21 (6.099)	0	17	3.00 (4.472)	0	13	0.35 (0.493)	0	1
PDD (63)	21.08 (7.208)	0	34	13.74 (3.702)	0	17	6.28 (3.804)	0	17	0.55 (0.501)	0	1

**Table 3:** Multicollinearity check and effect size concerning the BCFT total model.

	Tolerance	R^2^
BCFT	0.636	0.363
Age	0.682	0.317
Education	0.706	0.293
Sex	0.948	0.051

**Table 4:** Multicollinearity check and effect size concerning the BCFT copy model.

	Tolerance	R^2^
BCFT	0.843	0.156
Age	0.793	0.206
Education	0.739	0.260
Sex	0.946	0.053

**Table 5:** Multicollinearity check and effect size concerning the BCFT memory model.

	Tolerance	R^2^
BCFT	0.566	0.433
Age	0.608	0.391
Education	0.716	0.283
Sex	0.949	0.050

**Table 6:** Multicollinearity check and effect size concerning the BCFT recognition model.

	Tolerance	R^2^
BCFT	0.763	0.236
Age	0.741	0.258
Education	0.743	0.256
Sex	0.949	0.050

**Table 7:** Multinomial logistic regression concerning BCFT total score for each group.

Diagnostic Group *		B	SE	z	*p*	OR	95% CI	
							Lower	Upper
ADD	BCFT_T	−0.493	0.033	−14.78	<0.001	0.610	0.572	0.652
	AGE	0.144	0.016	8.74	<0.001	1.155	1.118	1.193
	EDUCATION	−0.116	0.031	−3.70	<0.001	0.890	0.837	0.946
	SEX	0.007	0.270	0.02	0.980	1.00	0.593	1.707
BV	BCFT_T	−0.495	0.037	−13.32	<0.001	0.609	0.566	0.655
	AGE	0.000	0.021	0.01	0.988	1.00	0.960	1.041
	EDUCATION	−0.084	0.044	−1.93	0.054	0.919	0.844	1.000
	SEX	−0.649	0.369	−1.76	0.079	0.522	0.253	1.076
CBD	BCFT_T	−0.537	0.043	−12.45	<0.001	0.584	0.536	0.635
	AGE	−0.109	0.027	−3.96	<0.001	0.897	0.850	0.946
	EDUCATION	−0.183	0.067	−2.72	0.006	0.832	0.729	0.949
	SEX	0.150	0.602	0.25	0.804	1.161	0.356	0.377
CVD/AD	BCFT_T	−0.489	0.035	−13.99	<0.001	0.613	0.572	0.656
	AGE	0.200	0.021	9.61	<0.001	1.221	1.173	1.272
	EDUCATION	−0.095	0.035	−2.75	0.006	0.909	0.849	0.973
	SEX	−0.839	0.304	−2.76	0.006	0.432	0.238	0.784
LBD	BCFT_T	−0.451	0.047	−9.54	<0.001	0.637	0.580	0.698
	AGE	0.206	0.038	5.41	<0.001	1.228	1.140	1.323
	EDUCATION	−0.103	0.057	−1.81	0.070	0.902	0.807	1.008
	SEX	−0.024	0.527	−0.05	0.963	0.976	0.347	0.274
NFV	BCFT_T	−0.419	0.053	−7.88	<0.001	0.657	0.592	0.729
	AGE	−0.070	0.033	−2.13	0.033	0.932	0.875	0.994
	EDUCATION	−0.130	0.081	−1.59	0.112	0.878	0.748	1.030
	SEX	−1.296	0.655	−1.98	0.048	0.273	0.075	0.988
PDD	BCFT_T	−0.344	0.036	−9.57	<0.001	0.709	0.660	0.760
	AGE	0.087	0.020	4.38	<0.001	1.090	1.049	1.133
	EDUCATION	−0.070	0.037	−1.87	0.061	0.932	0.866	1.003
	SEX	−0.915	0.320	−2.86	0.004	0.400	0.213	0.750
SV	BCFT_T	−0.506	0.041	−12.48	<0.001	0.602	0.556	0.652
	AGE	−0.056	0.026	−2.23	0.026	0.944	0.989	0.993
	EDUCATION	−0.219	0.057	−3.84	<0.001	0.803	0.718	0.898
	SEX	−0.776	0.464	−1.67	0.094	0.460	0.185	1.142

* The reference category is Control Group (healthy participants).

**Table 8:** Measurements with overall classification accuracy indexes.

BCFT Components	Overall Accuracy	McFadden’s R^2^	Nagelkerke R^2^
Total	72.41%	0.425	0.750
Copy	66.11%	0.314	0.633
Memory	73.28%	0.444	0.766
Recognition	67.48%	0.313	0.630

**Table 9:** Likelihood ratio test concerning all components of BCFT.

	df	x^2^	*p*
BCFT Total	8	689.87	<0.001
Age	8	270.66	<0.001
Education	8	23.14	0.003
Sex	8	32.88	<0.001
BCFT Copy	8	229.52	<0.001
Age	8	541.49	<0.001
Education	8	65.76	<0.001
Sex	8	30.77	<0.001
BCFT Memory	8	762.90	<0.001
Age	8	236.49	<0.001
Education	8	29.58	<0.001
Sex	8	34.45	<0.001
BCFT Recognition	8	220.12	<0.001
Age	8	481.19	<0.001
Education	8	78.33	<0.001
Sex	8	31.83	<0.001

**Table 10:** Per-class sensitivity—true-positive rate.

	Control	ADD	BV	CBD	CVD/AD	LBD	NFV	PDD	SV
Total	0.975	0.896	0.058	0.227	0.027	0.000	0.000	0.000	0.069
Copy	0.914	0.788	0.019	0.182	0.020	0.000	0.000	0.000	0.034
Memory	0.984	0.911	0.038	0.136	0.054	0.000	0.000	0.000	0.034
Recognition	0.929	0.825	0.000	0.000	0.020	0.000	0.000	0.000	0.000

**Table 11:** Per-class specificity—true-negative rate.

	Control	ADD	BV	CBD	CVD/AD	LBD	NFV	PDD	SV
Total	0.887	0.725	0.997	0.994	0.991	1.000	1.000	1.000	0.997
Copy	0.785	0.712	0.998	0.994	0.990	1.000	1.000	1.000	0.998
Memory	0.887	0.744	0.996	0.994	0.989	1.000	1.000	1.000	0.996
Recognition	0.782	0.730	0.999	0.996	0.990	1.000	1.000	1.000	0.998

## Data Availability

The data of the present analysis are stored on the Third-Age Day Center IASIS server and are available upon request to the mails of the project’s administrator.

## Abbreviations

The following abbreviations are used in this manuscript:

ADD	Alzheimer’s disease/Alzheimer’s disease dementia
PDD	Parkinson’s disease/Parkinson’s disease dementia
CBD	Corticobasal dementia
LBD	Lewy Body dementia
FTD	Frontotemporal dementia
FTD-BV	Frontotemporal dementia behavioral variant
NFV	Frontotemporal dementia progressive non-fluent aphasia
FTD-SV	Frontotemporal dementia semantic variant
CVD/AD	Cardiovascular dementia with Alzheimer’s disease
BCFT	Benson Complex Figure Test
ROCFT	Rey–Osterrieth Complex Figure Test

## References

[1] (2022). GBD 2019 Dementia Forecasting Collaborators. Estimation of the global prevalence of dementia in 2019 and forecasted prevalence in 2050: An analysis for the Global Burden of Disease Study 2019. Lancet Public Health.

[2] Livingston G., Huntley J., Liu K.Y., Costafreda S.G., Selbæk G., Alladi S., Ames D., Banerjee S., Burns A., Brayne C. (2024). Dementia prevention, intervention, and care: 2024 report of the Lancet standing Commission. Lancet.

[3] Agrawal M., Biswas A. (2015). Molecular diagnostics of neurodegenerative disorders. Front. Mol. Biosci..

[4] Scheltens P., De Strooper B., Kivipelto M., Holstege H., Chételat G., Teunissen C.E., Cummings J., van der Flier W.M. (2021). Alzheimer’s disease. Lancet.

[5] Castro-Alonso J.C., Atit K. (2019). Different abilities controlled by visuospatial processing. Visuospatial Processing for Education in Health and Natural Sciences.

[6] Olds J.J., Hills W.L., Warner J., Falardeau J., Alasantro L.H., Moster M.L., Egan R.A., Cornblath W.T., Lee A.G., Frishberg B.M. (2020). Posterior Cortical Atrophy: Characteristics from a Clinical Data Registry. Front. Neurol..

[7] Porsteinsson A.P., Isaacson R.S., Knox S., Sabbagh M.N., Rubino I. (2021). Diagnosis of Early Alzheimer’s Disease: Clinical Practice in 2021. J. Prev. Alzheimers Dis..

[8] Irish M., Piguet O., Hodges J.R., Hornberger M. (2014). Common and unique gray matter correlates of episodic memory dysfunction in frontotemporal dementia and Alzheimer’s disease. Hum. Brain Mapp..

[9] Karantzoulis S., Galvin J.E. (2011). Distinguishing Alzheimer’s disease from other major forms of dementia. Expert Rev. Neurother..

[10] Zhang X., Lv L., Min G., Wang Q., Zhao Y., Li Y. (2021). Overview of the complex figure test and its clinical application in neuropsychiatric disorders, including copying and recall. Front. Neurol..

[11] Salvadori E., Dieci F., Caffarra P., Pantoni L. (2019). Qualitative evaluation of the immediate copy of the Rey–Osterrieth complex figure: Comparison between vascular and degenerative MCI patients. Arch. Clin. Neuropsychol..

[12] Possin K.L., Laluz V.R., Alcantar O.Z., Miller B.L., Kramer J.H. (2011). Distinct neuroanatomical substrates and cognitive mechanisms of figure copy performance in Alzheimer’s disease and behavioral variant frontotemporal dementia. Neuropsychologia.

[13] Kemp J., Philippi N., Phillipps C., Demuynck C., Albasser T., Martin-Hunyadi C., Schmidt-Mutter C., Cretin B., Blanc F. (2017). Cognitive profile in prodromal dementia with Lewy bodies. Alzheimers Res. Ther..

[14] Bussè C., Mitolo M., Mozzetta S., Venneri A., Cagnin A. (2024). Impact of Lewy bodies disease on visual skills and memory abilities: From prodromal stages to dementia. Front. Psychiatry.

[15] Ferman T.J., Smith G.E., Boeve B.F., Graff-Radford N.R., Lucas J.A., Knopman D.S., Petersen R.C., Ivnik R.J., Wszolek Z., Uitti R. (2006). Neuropsychological differentiation of dementia with Lewy bodies from normal aging and Alzheimer’s disease. Clin. Neuropsychol..

[16] Murayama N., Ota K., Iseki E. (2024). The Bender Gestalt Test is useful for clinically diagnosing dementia with Lewy bodies: Analysis of its sensitivity, specificity, and clinical characteristics of the figure copy. Appl. Neuropsychol. Adult.

[17] Murayama N., Iseki E., Yamamoto R., Kimura M., Eto K., Arai H. (2007). Utility of the Bender Gestalt Test for differentiation of dementia with Lewy bodies from Alzheimer’s disease in patients showing mild to moderate dementia. Dement. Geriatr. Cogn. Disord..

[18] Beretta L., Carli G., Caffarra P., Perani D. (2022). Distinct brain dysfunctions underlying visuo-constructive deficit in DLB and AD. Brain Imaging Behav..

[19] Wang H.Y., Ren L., Yan Z., Zhou T., Liang Z. (2025). Neural basis of dysexecutive and visuospatial impairments in Parkinson’s disease with MCI: A task-based fNIRS study. npj Park. Dis..

[20] Betrouni N., Devignes Q., Bayot M., Derambure P., Defebvre L., Leentjens A.F., Delval A., Dujardin K. (2022). The frontostriatal subtype of mild cognitive impairment in Parkinson’s disease, but not the posterior cortical one, is associated with specific EEG alterations. Cortex.

[21] Lawson R.A., Williams-Gray C.H., Camacho M., Duncan G.W., Khoo T.K., Breen D.P., Barker R.A., Rochester L., Burn D.J., Yarnall A.J. (2021). Which Neuropsychological Tests? Predicting Cognitive Decline and Dementia in Parkinson’s Disease in the ICICLE-PD Cohort. J. Park. Dis..

[22] Sedeño L., Couto B., Garcia-Cordero I., Melloni M., Baez S., Sepúlveda J.P.M., Ibanez A. (2016). Brain network organization and social executive performance in frontotemporal dementia. J. Int. Neuropsychol. Soc..

[23] Laforce R.J. (2013). Behavioral and language variants of frontotemporal dementia: A review of key symptoms. Clin. Neurol. Neurosurg..

[24] Olney N.T., Spina S., Miller B.L. (2017). Frontotemporal Dementia. Neurol. Clin..

[25] Jiskoot L.C., Russell L.L., Peakman G., Convery R.S., Greaves C.V., Bocchetta M., Genetic Frontotemporal dementia Initiative (2023). The Benson Complex Figure Test detects deficits in visuoconstruction and visual memory in symptomatic familial frontotemporal dementia: A GENFI study. J. Neurol. Sci..

[26] Tartaglia M.C., Zhang Y., Racine C., Laluz V., Neuhaus J., Chao L., Weiner M. (2012). Executive dysfunction in frontotemporal dementia is related to abnormalities in frontal white matter tracts. J. Neurol..

[27] Wilson D., Le Heron C., Anderson T. (2021). Corticobasal syndrome: A practical guide. Pract. Neurol..

[28] Di Stasio F., Suppa A., Fabbrini A., Marsili L., Asci F., Conte A., Trebbastoni A., De Lena C., Berardelli A. (2018). Parkinsonism is associated with altered primary motor cortex plasticity in frontotemporal dementia-primary progressive aphasia variant. Neurobiol. Aging.

[29] Sengupta P., Ganguly J., Pal S., Ghosal M. (2019). Pattern of cognitive deficits in vascular dementia. Indian J. Med. Res..

[30] Freeman R.Q., Giovannetti T., Lamar M., Cloud B.S., Stern R.A., Kaplan E., Libon D.J. (2000). Visuoconstructional problems in dementia: Contribution of executive systems functions. Neuropsychology.

[31] Folstein M.F., Folstein S.E., McHugh P.R. (1975). “Mini-mental state”: A practical method for grading the cognitive state of patients for the clinician. J. Psychiatr. Res..

[32] Fountoulakis K.N., Tsolaki M., Chantzi H., Kazis A. (2000). Mini mental state examination (MMSE): A validation study in Greece. Am. J. Alzheimers Dis. Other Dement..

[33] Kouri N., Murray M.E., Hassan A., Rademakers R., Uitti R.J., Boeve B.F., Graff-Radford N.R., Wszolek Z.K., Litvan I., Josephs K.A. (2011). Neuropathological features of corticobasal degeneration presenting as corticobasal syndrome or Richardson syndrome. Brain.

[34] Jellinger K.A. (2023). Pathomechanisms of cognitive and behavioral impairment in corticobasal degeneration. J. Neural Transm..

[35] Gorno-Tempini M.L., Dronkers N.F., Rankin K.P., Ogar J.M., Phengrasamy L., Rosen H.J., Johnson J.K., Weiner M.W., Miller B.L. (2004). Cognition and anatomy in three variants of primary progressive aphasia. Ann. Neurol..

[36] Jellinger K.A., Korczyn A.D. (2018). Are dementia with Lewy bodies and Parkinson’s disease dementia the same disease?. BMC Med..

[37] Chang W.E., Chang C.H. (2022). Vascular Cognitive Impairment and Dementia. Continuum.

[38] Minoshima S., Cross D., Thientunyakit T., Foster N.L., Drzezga A. (2022). 18F-FDG PET Imaging in Neurodegenerative Dementing Disorders: Insights into Subtype Classification, Emerging Disease Categories, and Mixed Dementia with Copathologies. J. Nucl. Med..

